# Transactions of the American Medical Association

**Published:** 1854-11

**Authors:** 


*Transactions of the American Medical Association.* Instituted 1847. Vol. vii. New York: C. B. Norton. 1854.

We are happy to announce to our readers the appearance of this volume of the Trunsactions. During the last few years it has generally reached us about ten months after the date of the annual meeting of the society. We do not wish to be understood as attaching any blame to previous committees of publication, although in many instances it has been two or three months after our eastern exchanges were supplied, before western readers and western journals received it. It may be that our eastern friends received some new ideas in reference to the professional interests of the west at the last meeting in St. Louis.

The volume contains, in addition to the minutes of the Seventh Annual Meeting, the address of Dr. Parsons, Vice-President, the report of the Committee on Medical Education, the report of the Committee on the Epidemics of Kentucky and Tennessee, on Erysipelas, by R. S. Holmes, M.D., of St. Louis; on the Medicinal and Toxicological properties of the Cryptogamic Plants of the United States, by Dr. Proctor, of Charleston, S. C.; Report on the Epidemics of Ohio, Indiana and Michigan, for the years 1852—3; Report on the Epidemics of Louisiana, Arkansas, and Texas in the year 1853; the prize essay on a new method of Treating Ununited Fractures and certain Deformities of the Osseous System, by Dr. D. Brainard, of Chicago; report on the Norwalk Disaster, and the reports of the Committee of Publication, Catalogue of the Officers, and permanent members and index.

We shall give analyses of the most interesting of these papers in future numbers of the Journal. The mechanical execution of the volume is good and does credit both to the committee and the publishers. We advise those of our readers who wish to secure a copy for their libraries to send in their orders soon to Dr. Pliny Earle, New York Chairman of the Committee of Publication.

